# Hypoxia Affects Nitrogen Uptake and Distribution in Young Poplar (*Populus* × *canescens*) Trees

**DOI:** 10.1371/journal.pone.0136579

**Published:** 2015-08-26

**Authors:** Bin Liu, Heinz Rennenberg, Jürgen Kreuzwieser

**Affiliations:** Chair of Tree Physiology, Institute of Forest Sciences, University of Freiburg, Freiburg, Germany; University of Vigo, SPAIN

## Abstract

The present study with young poplar trees aimed at characterizing the effect of O_2_ shortage in the soil on net uptake of NO_3_^-^ and NH_4_^+^ and the spatial distribution of the N taken up. Moreover, we assessed biomass increment as well as N status of the trees affected by O_2_ deficiency. For this purpose, an experiment was conducted in which hydroponically grown young poplar trees were exposed to hypoxic and normoxic (control) conditions for 14 days. ^15^N-labelled NO_3_^-^ and NH_4_^+^ were used to elucidate N uptake and distribution of currently absorbed N and N allocation rates in the plants. Whereas shoot biomass was not affected by soil O_2_ deficiency, it significantly reduced root biomass and, consequently, the root-to-shoot ratio. Uptake of NO_3_^-^ but not of NH_4_^+^ by the roots of the trees was severely impaired by hypoxia. As a consequence of reduced N uptake, the N content of all poplar tissues was significantly diminished. Under normoxic control conditions, the spatial distribution of currently absorbed N and N allocation rates differed depending on the N source. Whereas NO_3_^-^ derived N was mainly transported to the younger parts of the shoot, particularly to the developing and young mature leaves, N derived from NH_4_^+^ was preferentially allocated to older parts of the shoot, mainly to wood and bark. Soil O_2_ deficiency enhanced this differential allocation pattern. From these results we assume that NO_3_^-^ was assimilated in developing tissues and preferentially used to maintain growth and ensure plant survival under hypoxia, whereas NH_4_^+^ based N was used for biosynthesis of storage proteins in bark and wood of the trees. Still, further studies are needed to understand the mechanistic basis as well as the eco-physiological advantages of such differential allocation patterns.

## Introduction

As an important constituent of amino acids, proteins, nucleic acids, N-based osmo-protectants and defence compounds, nitrogen (N) is an essential major nutrient of plants. Important N compounds taken up by plant roots are the inorganic NO_3_^-^ and NH_4_^+^ [1] as well as organic N such as amino acids [2–4]. The concentrations of the different N compounds in forest soils vary considerably [5, 6] and highly depend on processes such as leaching or volatilization of N, but also on microbial processes using N compounds as substrates, including immobilisation, mineralisation, nitrification and denitrification [7]. Such processes are strongly influenced by environmental conditions [8]. For example, soil O_2_ deficiency favours denitrification which leads to reduced abundance of NO_3_^-^ but to increased NH_4_^+^ concentrations in the soil [9, 10], whereas in O_2_ rich soils nitrification dominates over denitrification leading to the formation of NO_3_^-^ from NH_4_^+^ [11]. It is well understood that N uptake by roots is strongly affected by the abundance of other N compounds, as, for example, reduced N such as NH_4_^+^ or amino acids inhibit NO_3_^-^ net uptake of coniferous and deciduous trees [4, 12–14].

Waterlogging and flooding are common environmental constrains leading to O_2_ deficiency in soils. Whereas energy metabolism is not limited under normoxia, O_2_ availability below 30 kPa (“critical O_2_ pressure” [15]) limits respiratory ATP generation under hypoxia. In contrast, under anoxia the absence of O_2_ allows only insignificant ATP generation by respiration [16]. Consequently, waterlogging and flooding can cause an energy crisis in the plant tissues affected [17, 18]. To maintain energy metabolism, hypoxic tissues switch from respiration to fermentative processes, mainly alcoholic fermentation [16, 19, 20]. However, fermentation is an energetically inefficient pathway because it yields only 2 molecules ATP per molecule glucose consumed as compared to 38 molecules ATP formed in mitochondrial respiration. Analysis of the plant transcriptome has revealed that under O_2_ deficiency plants minimize energy consumption by slowing down ATP demanding processes including growth, biosynthesis of polymers and active transport processes [21–24]. As a major nutrient, N uptake comprises ca. 80% of all nutrients absorbed by roots from the soil [25] and, therefore, constitutes a strong energy sink [26]. Particularly, the active uptake of the quantitatively important NO_3_^-^ which mechanistically occurs via proton symport, strongly depends on the ATP consuming maintenance of the proton gradient across the plasma membrane. In contrast, NH_4_^+^ uptake is energetically favored, because it occurs thermodynamically “downhill” at concentrations above 200–500 μM. At lower concentrations, it is considered secondarily active, e.g. occurring through ATP-dependent NH_4_^+^ pumps or via NH_4_^+^ /H^+^ cotransport [18, 27, 28, 29]. Consequently, root uptake of NO_3_^-^ is impaired by soil O_2_ deprivation, whereas the energetically more advantageous NH_4_^+^ uptake seems to be less affected [30, 31]. However, in such studies NO_3_^-^ and NH_4_^+^ were supplied individually as sole N source, and it is still unknown, how soil O_2_ deficiency affects NO_3_^-^ and NH_4_^+^ uptake, if both nutrients are supplied in combination.

NO_3_^-^ taken up by the roots is channelled into assimilatory NO_3_^-^ reduction in the roots of many tree species, which is in contrast to herbaceous plants assimilating NO_3_^-^ mainly in green tissues [32, 33]. Thus, in trees, reduced N, mainly as amino acids, is transported in the transpiration stream to the leaves, and is further distributed and plant-internally cycled [12, 34, 35]. Such cycling seems to be a tree specific feature, which ensures supply of reduced N to N demanding tissues [36]. Also in poplar, NO_3_^-^ assimilation can occur in the roots; however, if NO_3_^-^ reduction capacity of roots is exceeded because of high soil NO_3_^-^ availability, the surplus NO_3_^-^ is transported to the shoot and assimilated in the leaves [37, 38]. NO_3_^-^ assimilation needs the sequential action of the enzymes NO_3_^-^ reductase (NR, EC 1.6.6.1) forming NO_2_^-^, and NO_2_^-^ reductase (NiR, EC 1.7.2.1) generating NH_4_^+^ in an energy demanding manner [39]. NH_4_^+^ is then used for the biosynthesis of organic N in form of amino acids by the glutamine synthetase/glutamine-oxoglutarate aminotransferase (GS/GOGAT) system (GS, EC 6.3.1.2/ Fd-ferredoxin-GOGAT, EC 1.4.7.1; NADH-GOGAT, EC 1.4.1.14) [40–41] and subsequent transamination reactions [42].

Soil O_2_ deficiency not only affects plant N uptake but also N metabolism at the physiological and the transcriptomic level [23]. Consistently, altered concentrations of amino acids, proteins and N-containing pigments have been observed in response to flooding [30, 43] with consequences for major plant processes such as photosynthesis [44]. In contrast, effects of soil O_2_ deficiency on plant-internal distribution of N is scarcely studied. Impacts of soil O_2_ deprivation on root-to-shoot transport of N-compounds can be assumed due to the often strongly slowed-down transpiration stream following soil hypoxia [45]. Because of the strong energy dependence of NO_3_^-^ uptake and assimilation as well as phloem transport of reduced N, impairment of these processes under O_2_ depletion must be assumed. The present study was performed to test the hypotheses that (i) net uptake of N, particularly of NO_3_^-^, by roots of young Gray poplar trees is affected by soil O_2_ deficiency leading to reduced biomass formation and total N contents in the trees, that (ii) the trees’ transpiration stream will be slowed down in response to soil O_2_ shortage, which will (iii) cause reduced allocation of N from roots to the leaves. As NO_3_^-^ assimilation will be strongly reduced under conditions of O_2_ limitation, we hypothesize differential effects on the allocation of N derived from NO_3_^-^ and NH_4_^+^. To test these hypotheses, we elucidated the spatial distribution of the currently absorbed N as affected by soil O_2_ deficiency. We exposed the roots of poplar trees to normoxic and hypoxic conditions, supplied them with ^15^N-labelled NO_3_^-^ and NH_4_^+^ and followed the allocation and distribution of ^15^N through the whole plant.

## Materials and Methods

### Plant material and growth conditions

The present experiments were performed with four months old Gray poplar (*Populus x canescens* clone INRA 717 1-B4) seedlings, which were micro-propagated as described earlier [46]. Four weeks old poplar cuttings cultivated in sterile culture tubes were transplanted to plastic pots (13 cm × 13 cm × 13 cm) containing sand (Glaser Trockensand GmbH, Malsch, Germany) treated with 0.15% fungicide solution (Proplant, Dr. Stählem GmbH, Stade, Germany) to minimize growth of pathogenic fungi. Plantlets were supplied regularly with distilled water; in addition, they were fertilized twice a week with 200 ml 25% modified Hoagland solution [47] consisting of 0.6 mM KNO_3_, 1.3 mM Ca(NO_3_)_2_ × 4 H_2_O, 0.3 mM MgSO_4_, 1.5 mM MgCl_2_, 0.25 mM KH_2_PO_4_, 2.3 μM MnCl_2_ × 4 H_2_O, 10 μM H_3_BO_3_, 0.08 μM CuCl_2_ × 4 H_2_O, 0.2 μM ZnCl_2_, 0.2 μM Na_2_MoO_2_ × 4 H_2_O, 0.04 μM CoCl_2_ × 6 H_2_O, 22.5 μM Na-EDTA, 22.5 μM FeCl_2_ (pH 5.5). The plants were grown under long day condition (16h light/8 h dark) at a temperature of 22±5°C for 4 months in a greenhouse.

### Experimental setup and protocol for introducing hypoxia

For experiments the seedlings were carefully taken out of the pots. After removing the sand from roots, each seedling was transferred into an amber glass bottle, which was filled with 1 L Hoagland nutrient solution; trees were adapted to the hydroponic environment for three days. During this time, the solutions were aerated with ambient air by means of air pumps (Schemel & Goetz GmbH & Co KG, Offenbach a. M., Germany). To avoid evaporation of water from the nutrient solution, all bottles were tightly sealed with parafilm (Bemis Company, Inc., Neenah, USA). Hypoxia was implemented for 14 days by stopping aeration. As a consequence, the O_2_ concentrations in the nutrient solution dropped to constant levels of 0.007±0.006 mg L^-1^, whereas it remained constantly between 7–8 mg L^-1^ in the aerated solutions as indicated by O_2_ determination with an O_2_ microsensor (Microx TX2; PreSens, Regensburg, Germany).

### Transpiration rates

Transpiration rates of the seedlings were calculated by weighing the water loss from the bottles containing the nutrient solution every two to three days until the 11^th^ day of soil O_2_ deficiency.

### ^15^N labelling and plant harvest

To study NO_3_^-^ and NH_4_^+^ net uptake and the distribution of currently absorbed N, ^15^N-labelling experiments were performed with 48 seedlings whose total root systems were exposed to either normal or reduced O_2_ availability for 14 days. For this purpose, the non-labelled solutions were completely removed from the bottles and replaced by nutrient solutions containing either ^14^NH_4_Cl and K^15^NO_3_ or ^15^NH_4_Cl and K^14^NO_3_ (n = 10–12) at final concentrations of 2.0 mM N (10%-atom ^15^N-abundance). Before adding these solutions, they were aerated (normoxia) or bubbled with N_2_ gas, in order to maintain the O_2_ concentrations in the bottles containing the trees. Natural ^15^N-abundances were used for correcting ^15^N labelling of each plant tissue. For this purpose, in parallel with the labelling experiment, trees exposed to normoxic or hypoxic conditions (8 trees per treatment) were supplied with non-labelled nutrient solutions. Two hours after exposure to the labelled nutrient solutions, poplar seedlings were harvested. For this purpose, each plant was carefully taken out from the bottle; the root part was immediately washed with tap water and then washed again with demineralized water; the whole seedlings were divided into four main sections: (1) the top 40 cm representing the developing part of the shoot, (2) the middle 40 cm section representing the younger mature part of the shoot, (3) the bottom section, ca. 50 cm in length, representing the older mature shoot section, and (4) the root section. Each shoot section was further divided into leaf, petiole, wood and bark, and the root section was further separated into coarse roots (>2 mm diameter) and fine roots (≤2 mm diameter). All plant parts were weighed and oven dried at 60°C until weight constancy. Dry samples were weighed and stored at room temperature until ^15^N analysis.

### Analysis of total N and ^15^N contents

Total N contents and ^15^N-abundances in different plant tissues (fine and coarse roots; leaves, petioles, wood and bark from the top 40 cm, middle 40 cm and lowest 50 cm shoot sections) were analyzed by a C/N 2500 analyzer (CE Instruments, Milan, Italy) coupled to a mass spectrometer (IR-MS, Finnigan MAT GmbH, Bremen, Germany). All dry tissues were well powdered and homogenized by a ball mill (MM 400, Retsch GmbH, Haan, Germany). Depending on the tissue to be analyzed, aliquots of 2.0 to 6.0 mg were weighed into tin capsules (IVA Analysentechnik, Meerbusch, Germany) which were burned into gases in the element analyzer and further analyzed in the mass spectrometer. For the calculation of total N contents in different tissues, plants exposed to hypoxia and treated with ^15^NO_3_^-^/^14^NH_4_^+^ (10–12 biological replicates) were combined with plants treated with ^14^NO_3_^-^/^15^NH_4_^+^ (10–12 biological replicates) because exposure to these differently labelled N sources cannot influence the total N content; thus, for this approach 20–24 biological replicates were used.

### Calculation of ^15^N distribution, rates of ^15^N allocation and ^15^NO_3_^-^ /^15^NH_4_^+^ uptake

The ^15^N allocation rates into different tissues were calculated with Eq (1),

NAR (nmol g−1DW h−1)=ΔN15tissue⋅[N]⋅DWtotal⋅1010DWtissue⋅Δt⋅M(N) (1)

where NAR is the NO_3_^-^ and NH_4_^+^ allocation rate (nmol g^-1^ DW h^-1^); Δ^15^N_tissue_ the difference of ^15^N abundance (% of total N) of different tissues from ^15^N-treated plant and non-labelled control plants (natural ^15^N abundance); [N] the total N concentration (g N g^-1^ DW); DW_total_ the total dry weight (g); DW_tissue_ the tissue dry weight (g); Δt the incubation time (h); M (N) the molecular weight of ^15^N (15 g mol^-1^). The calculation of total ^15^N per tissue was based on the specific ^15^N contents of the labelling solution and tissue biomass. Total ^15^N per plant was calculated by summing up the total ^15^N contents in all tissues.

NO_3_^-^ or NH_4_^+^ uptake rates were calculated from the total ^15^N accumulation in the plants during the incubation period and were based on fresh weight of fine roots. For the calculation of NO_3_^-^ and NH_4_^+^ uptake rates, eqs (2) and (3) were used,

NUR (nmol g−1FW h−1)=ΔN15plant⋅[N]⋅DWtotal⋅1010FWfr⋅Δt⋅M(N) (2)

N15plant=∑n=13(N 15leaf.n+N15petiole.n+N15wood.n+N15bark.n)+N15fr+N15cr (3)

where in Eq (2) NUR is the specific NO_3_^-^ or NH_4_^+^ net uptake rate (nmol g^-1^ FW h^-1^); Δ^15^N_plant_ the difference of ^15^N abundance (% of total N) of whole plants from ^15^N-treated plant and non-labelled control plants (natural ^15^N abundance); [N] the total N concentration (g N g^-1^ DW); DW_total_ the total dry weight (g); FW_fr_ the fresh weight of fine roots (g); Δt the incubation time (h); M (N) the molecular weight of ^15^N (15 g mol^-1^). In Eq (3) ^15^N_plant_ is total ^15^N abundance (atom percentage) in the whole plants; ^15^N_leaf.n_, ^15^N_petiole.n_, ^15^N_wood.n_ and ^15^N_bark.n_ the ^15^N abundances (atom percentage) in the respective tissues from three different positions of the shoots, *i*.*e*. top 40 cm, middle 40 cm and lowest 50 cm; ^15^N_fr_ and ^15^N_cr_ are ^15^N abundances (atom percentage) in fine roots and coarse roots, respectively.

### Statistical analysis

Data were tested for normality (Shapiro-Wilk test) and equality of variances. If required, we applied a logarithmic transformation (common logarithm) on the raw data. Significant differences between controls and hypoxia treated plants were determined using one-way analysis of variance (ANOVA) and Student’s *t-test*. When the normality test failed, the Kruskal-Wallis one-way ANOVA on ranks and the Mann-Whitney rank sum test were used instead. All statistical analyses were performed using Sigmaplot 11.0 (Systat Software GmbH, Erkrath, Germany).

## Results

### Growth parameters and transpiration

**Fig 1:**
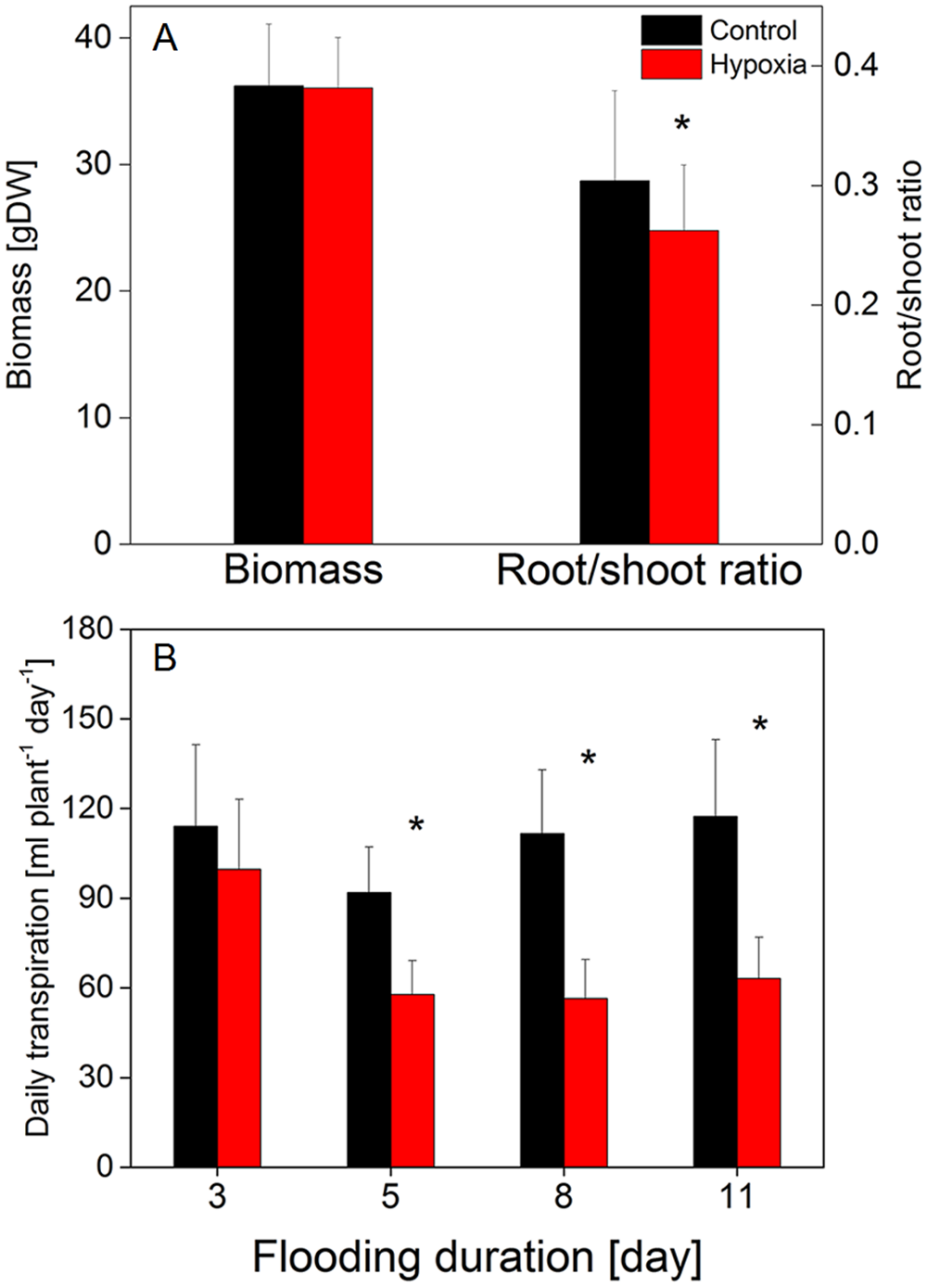
Effect of soil O_2_ deficiency on biomass accumulation and daily transpiration of young poplar trees. Four months old, hydroponically grown poplar trees were exposed to either normoxic or hypoxic conditions. After 14 days of treatment the plants were harvested, oven dried and the dry weights determined. Root-to-shoot ratios were calculated for each plant. In addition, daily transpiration rates were determined. Data shown are means ± SD of 10–12 biological replicates per treatment. Statistically significant differences at p< 0.05 between hypoxic and normoxic plants were calculated by Student’s t-test and are shown by asterisk.

Poplar trees exposed to soil O_2_ deficiency showed significantly decreased fine root biomass formation compared to trees grown at sufficient O_2_ supply (Table 1). In contrast, most of the other plant organs did not show significant differences depending on soil O_2_ availability. As a consequence, total biomass of poplar trees was the same under both treatments, but the root-to-shoot ratio decreased under soil O_2_ deficiency (Fig 1). Rates of transpiration significantly decreased under hypoxia beginning from the 5^th^ day of the treatment (Fig 1).

**Table 1:** Effect of soil O_2_ deficiency (hypoxia) on biomass (g DW) of poplar plants.

*Shoot*	Top 40 cm		Mid 40 cm		Lowest 50 cm		Total	
	Normoxia	Hypoxia	Normoxia	Hypoxia	Normoxia	Hypoxia	Normoxia	Hypoxia
Leaf	2.43±0.33	2.37±0.25	2.61±0.50	2.79±0.63	5.70±1.37	5.53±1.04	10.75±1.50	10.69±1.43
Petiole	0.22±0.04	0.23±0.04	0.32±0.04	0.34±0.05	0.74±0.16	0.69±0.18	1.28±0.19	1.26±0.20
Bark	0.46±0.05	0.46±0.05	**0.72±0.23**	**0.79±0.18**	3.40±0.53	3.66±0.57	4.53±0.65	4.70±1.00
Wood	0.60±0.21	0.58±0.09	1.52±0.13	1.61±0.26	9.12±1.32	9.39±1.44	11.18±1.39	11.57±1.37
*Roots*	Fine roots		Coarse roots				Total	
	Normoxia	Hypoxia	Normoxia	Hypoxia			Normoxia	Hypoxia
	**3.49±1.41**	**2.93±1.01**	4.97±1.31	4.69±1.06			8.46±2.44	7.62±1.72

During harvest the trees were divided into leaf, petiole, bark, wood, fine roots and coarse roots. The shoot was separated into the top 40 cm, middle 40 cm and bottom 50 cm Data shown are means ± SD of 22–24 biological replicates. The differences between plants exposed to normal O_2_ supply (normoxia) and reduced soil O_2_ supply (hypoxia) were tested by Student’s t-test at p<0.05; significant differences are indicated by bold.

### Soil O_2_ deficiency affects N content in plant tissues

**Fig 2:**
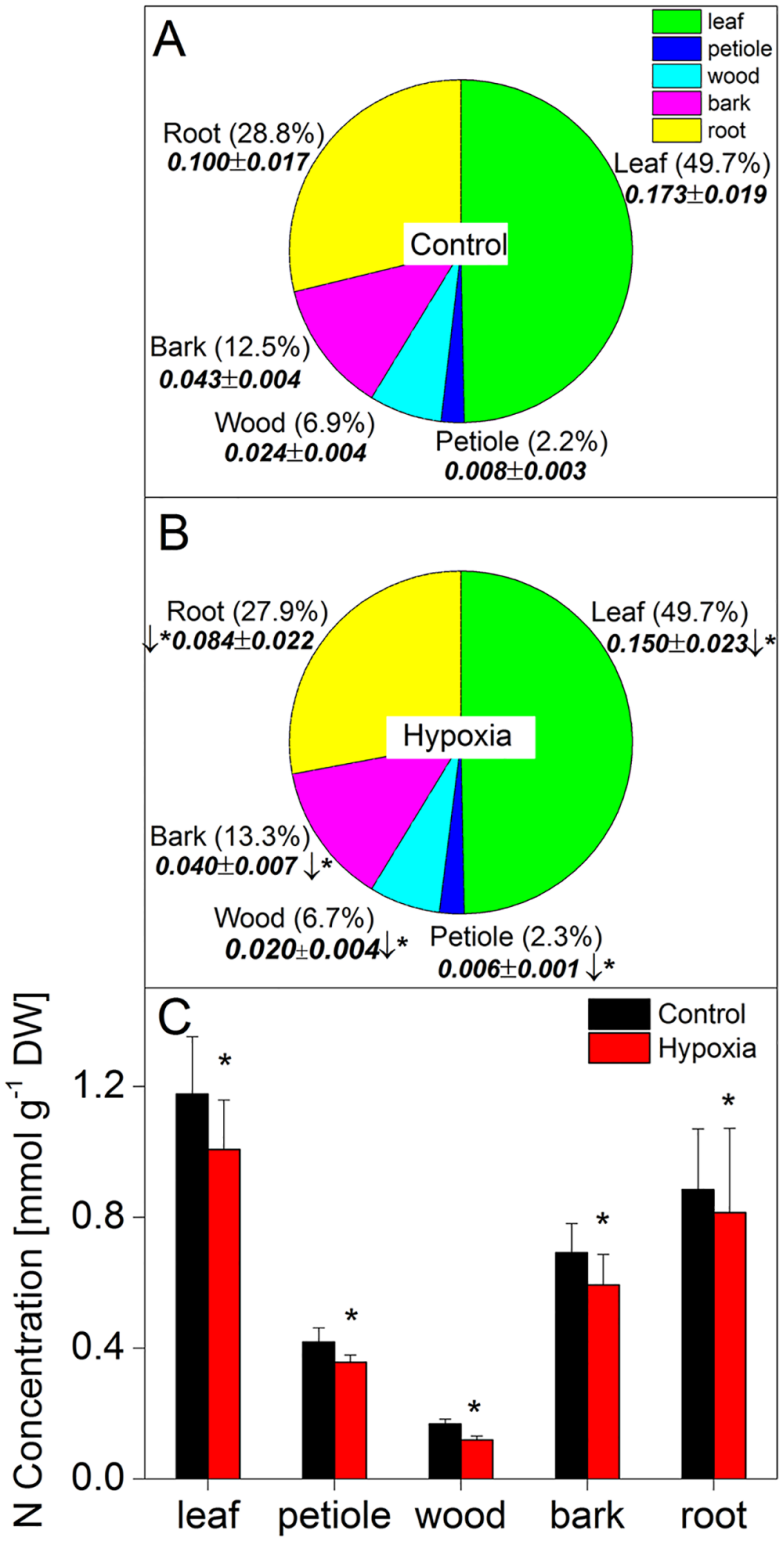
Effects of hypoxia on the total N contents (A, B) and N concentrations (C) in organs of young poplar trees. Trees were exposed to either normoxia (A) or hypoxia (B) for 14 days. After the treatment period, the plants were harvested, divided into the different parts, oven dried and after homogenization the total N contents (g organ^-1^), relative portion of N (% of total N in plant) and concentrations (mmol g^-1^ DW) determined. Data shown are means ± SD of 22–24 biological replicates. Statistically significant differences at p<0.05 between plants exposed to either hypoxia or normoxia were tested by Student’s t-test and are indicated by asterisks.

We assessed total N contents in different above- and belowground parts of the poplar trees studied (Fig 2). Soil O_2_ shortage significantly reduced the total N contents in all plant organs investigated. This effect was most pronounced in leaves and roots, where total N content decreased from 0.17±0.02 (normoxia) to 0.15±0.02 (hypoxia) g plant^-1^ and 0.10±0.02 (normoxia) to 0.08±0.02 (hypoxia) g plant^-1^, respectively (Fig 2A and 2B). The relative distribution of N, however, did not change due to O_2_ deficiency. Leaves, for example, contained ca. 50% of total plant N independent on the treatment. Roots contained ca. 29% of total plant N, bark tissue ca. 13%, wood tissue ca.7% and the petioles ca. 2% of total plant N (Fig 2A and 2B). When expressed on a dry weight basis, hypoxia also resulted in significantly decreased N concentrations in all organs (Fig 2C). The N concentrations of the different above-ground plant organs depended on the position on the shoot (Table 2) with the uppermost plant parts consistently containing the highest N concentrations.

**Table 2:** N contents [mmol g^-1^ DW] in poplar plants exposed to normoxia or hypoxia for 14 days.

*Shoot*	Top 40 cm		Mid 40 cm		Lowest 50 cm		Total	
	Normoxia	Hypoxia	Normoxia	Hypoxia	Normoxia	Hypoxia	Normoxia	Hypoxia
Leaf	**1.59±0.28**	**1.34±0.20**	**1.14±0.18**	**0.90±0.18**	1.02±0.16	0.94±0.22	**1.18±0.16**	**1.01±0.15**
Petiole	**0.59±0.11**	**0.48±0.08**	0.40±0.04	0.48±0.29	**0.38±0.08**	**0.34±0.03**	**0.42±0.06**	**0.40±0.07**
Bark	**0.80±0.16**	**0.59±0.13**	**0.54±0.11**	**0.43±0.10**	**0.70±0.09**	**0.62±0.09**	**0.69±0.09**	**0.59±0.09**
Wood	**0.49±0.11**	**0.36±0.12**	**0.16±0.03**	**0.12±0.03**	**0.13±0.03**	**0.11±0.02**	**0.15±0.03**	**0.12±0.02**
*Roots*	Fine roots		Coarse roots				Total	
	Normoxia	Hypoxia	Normoxia	Hypoxia			Normoxia	Hypoxia
	1.48±0.45	1.38±0.37	**0.49±0.08**	**0.39±0.07**			**0.88±0.19**	**0.76±0.14**

During harvest the trees were divided into leaf, petiole, bark, wood, fine roots and coarse roots. The shoot was separated into the top 40 cm, middle 40 cm and bottom 50 cm Data shown are means ± SD of 22–24 biological replicates. The differences between plants exposed to either hypoxia or normoxia were tested by Student’s *t-test* at p< 0.05; significant differences are indicated by bold.

### O_2_ shortage affects ^15^NO_3_^-^ but not ^15^NH_4_^+^ net uptake

**Fig 3:**
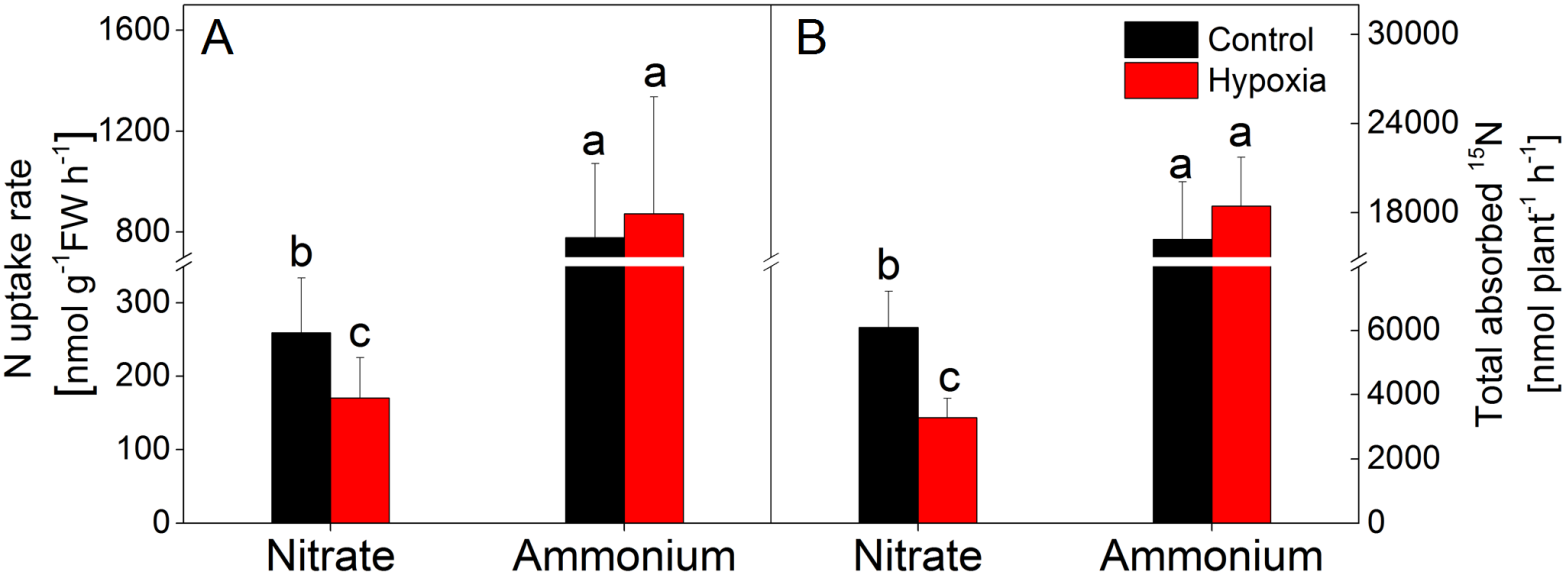
Effects of hypoxia on ^15^NO_3_^-^ and ^15^NH_4_^+^ uptake rate. After a treatment period of 14 days, the poplar seedlings were supplied with either ^14^NH_4_Cl and K^15^NO_3_ or ^15^NH_4_Cl and K^14^NO_3_ at final N concentrations of 2.0 mM and incubated for 2 h. Trees were then harvested, ^15^N contents analyzed in dried tissues and N uptake rates calculated as described in materials and methods. Data shown are means ± SD of 10–12 biological replicates. The differences between hypoxic and normoxic control plants were calculated by LSD under ANOVA. Different lower case letters indicate statistical differences at p<0.05 between control and hypoxia treated poplar trees supplied with ^15^NO_3_^-^ or ^15^NH_4_^+^.

N uptake rates were determined after application of NH_4_Cl in combination with KNO_3_, where either the NH_4_^+^ or the NO_3_^-^ was labelled with ^15^N. At normal O_2_ supply, NH_4_^+^ uptake (778±293 nmol g^-1^ FW h^-1^) was about 3-times higher than NO_3_^-^ uptake (259±75 nmol g^-1^ FW h^-1^) (Fig 3). Soil O_2_ deficiency did not influence the uptake of NH_4_^+^, however, NO_3_^-^ uptake was significantly decreased (170±55 nmol g^-1^ FW h^-1^) under these conditions (Fig 3A). A very similar pattern with reduced NO_3_^-^ but unaffected NH_4_^+^ uptake was obtained if the N absorption at the whole plant level was calculated (Fig 3B).

### Hypoxia alters the root-to-shoot distribution of N currently taken up

**Fig 4:**
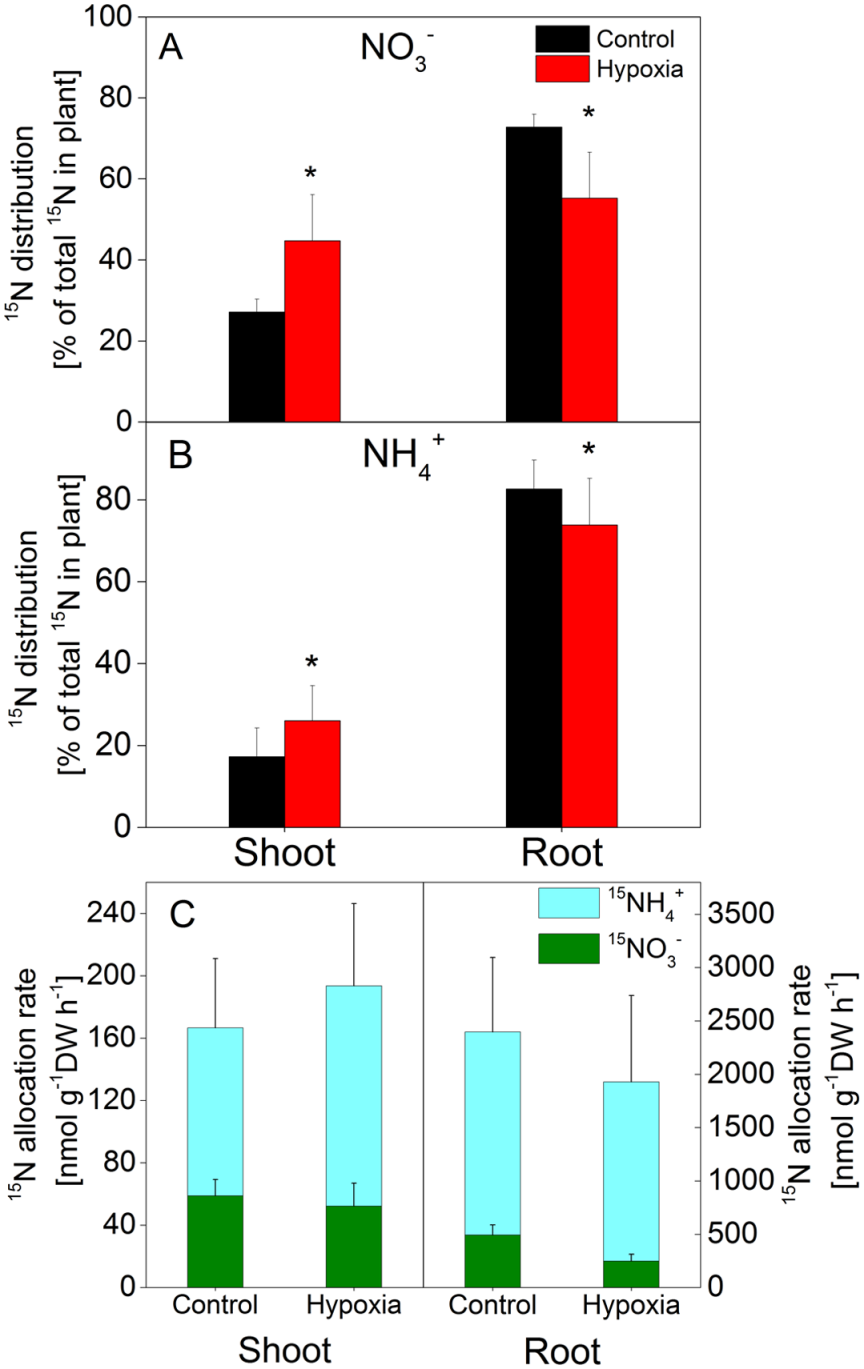
Effect of hypoxia on the root—shoot distribution (A, B) and ^15^N allocation rates (C) in young poplar trees. Young poplar trees were kept for 14 days under normoxic (A) or hypoxic (B) conditions and then supplied with ^15^NO_3_^-^ or ^15^NH_4_^+^ as described in legend of Fig 3. ^15^N contents were determined in all plant organs and data used to calculate the parameters shown. Data shown are means ± SD of 10–12 biological replicates. The differences between normoxia and hypoxia treated plants were tested by Student’s t-test at p< 0.05.

To investigate into which plant parts the currently absorbed N was distributed, the total ^15^N detected in roots and the shoot was assessed. The major parts of ^15^N derived from NO_3_^-^ (*i*.*e*. ^15^NO_3_^-^N) or NH_4_^+^ (*i*.*e*. ^15^NH_4_^+^-N) taken up by the trees, was found in the roots (Fig 4A). Soil O_2_ deficiency caused a significant decrease in root incorporated ^15^N. Under these conditions only 55±11% of the ^15^N absorbed as ^15^NO_3_ remained in the roots compared to 73±3% under normal O_2_ supply. Consequently, the portion of totally absorbed ^15^NO_3_^-^N which was transported from the roots to the shoot increased from 27±3% to 44±11%.

O_2_ shortage also reduced the portion of ^15^NH_4_^+^-N which remained in the roots from 83±7% to 74±9% of the total ^15^N taken up (Fig 4B). Consequently, similar to NO_3_, a significantly higher portion of the ^15^N absorbed by the roots was allocated to the shoot under these conditions. Irrespective of these changes, the allocation rates of ^15^NO_3_^-^N and ^15^NH_4_^+^-N from roots to the shoot were unaffected by hypoxia (Fig 4C). Noteworthy, the allocation rates to the roots result from the balance of the rates of N net uptake and N transport from roots to shoot.

### ^15^NO_3_^-^N accumulates in developing leaves but ^15^NH_4_^+^-N mainly in older sections of wood and bark

To study the distribution of currently absorbed ^15^N, all plant organs were separately analyzed for their ^15^N content. Under normal O_2_ supply, the developing leaves and the younger mature leaves were strong sinks for ^15^NO_3_^-^N (Table 3). In the lowest, older parts of the plant, ^15^NO_3_^-^N was mainly detected in the wood. This pattern of distribution was enhanced under hypoxia, where the portions significantly increased from 3.3±1.1% to 6.3±3.3% (^15^N in youngest leaves) and from 11±2% to 22±13% (^15^N in oldest wood). Around 20% of the ^15^NO_3_^-^N taken up, accumulated in the coarse roots independent of the treatment. Fine roots of hypoxically treated plants contained ca. 36% less ^15^N than trees kept under normoxia.

**Table 3:** Distribution of absorbed ^15^N (% of total ^15^N taken up per plant) in poplar plants kept under normoxia or hypoxia.

	Top 40 cm		Mid 40 cm		Lowest 50 cm		Sum	
	normoxia	hypoxia	normoxia	hypoxia	normoxia	hypoxia	normoxia	hypoxia
^*15*^ *NO*_*3*_^*-*^ *supplied*								
Leaf	**3.28±1.11**	**6.32±3.30**	**3.92±1.55**	**6.54±2.95**	2.82±1.99	2.90±1.19	**10.02±2.92**	**15.76±5.80**
Petiole	0.19±0.20	0.28±0.30	0.41±0.30	n.d.	1.17±0.33	0.88±0.58	1.54±0.46	1.09±0.76
Bark	0.15±0.07	0.30±0.31	0.32±0.14	0.42±0.28	2.45±0.87	2.78±1.31	2.92±0.89	3.50±1.46
Wood	0.31±0.24	0.34±0.13	**1.45±0.84**	**2.95±1.66**	10.92±2.20	21.62±12.85	**12.69±1.97**	**24.90±12.53**
Sum	**3.83±1.12**	**6.96±3.02**	**5.98±2.06**	**10.02±4.05**	**17.37±2.02**	**27.77±12.60**	**27.17±3.14**	**44.75±11.36**
Coarse roots	20.70±7.76	21.89±4.48						
Fine roots	**52.13±7.42**	**33.36±10.53**						
Sum	**72.83±3.14**	**55.25±11.36**						
^*15*^ *NH*_*4*_^*+*^ *supplied*								
Leaf	0.62±0.45	0.22±0.13	0.51±0.55	0.33±0.35	1.56±1.13	1.75±1.46	2.68±1.92	2.13±1.79
Petiole	0.14±0.22	0.13±0.14	0.19±0.10	0.19±0.22	0.62±0.21	1.18±1.22	0.94±0.40	1.47±1.36
Bark	0.22±0.37	0.13±0.21	0.22±0.16	0.22±0.20	**3.01±1.13**	**6.69±4.03**	**3.45±1.45**	**7.04±3.95**
Wood	0.33±0.31	0.24±0.26	0.57±0.48	1.25±1.45	**9.31±1.18**	**13.95±6.40**	**10.21±4.63**	**15.44±6.43**
Sum	1.30±1.15	0.56±0.64	1.49±1.02	1.95±1.56	**14.50±5.37**	**23.57±7.70**	**17.29±7.02**	**26.08±8.59**
Coarse roots	17.73±3.50	16.25±6.63						
Fine roots	64.98±8.73	57.67±10.72						
Sum	**82.71±7.02**	**73.92±8.59**						

Data shown are means ± SD of 10–12 biological replicates; statistically significant differences at p<0.05 between normoxic controls and hypoxically treated plants were calculated by Student’s *t-test* and are indicated by bold.

In contrast to ^15^N derived from ^15^NO_3_^-^, where the young leaves were a major sink of ^15^N, the ^15^NH_4_^+^-N was mainly found in wood, bark, petioles and leaves of the lowest parts of the trees (Table 3). This pattern was further enhanced if the trees were kept under conditions with reduced O_2_ supply. The coarse roots contained somewhat less ^15^NH_4_^+^-N (ca. 17% of the total ^15^NH_4_^+^ taken up) than ^15^N from ^15^NO_3_^-^. With around 60% of the total ^15^NH_4_^+^ absorbed by the plant, the fine roots accumulated the highest ^15^N content independent of the O_2_ concentrations of the nutrient solution.

### Effects of soil O_2_ deficiency on N allocation rates

**Fig 5:**
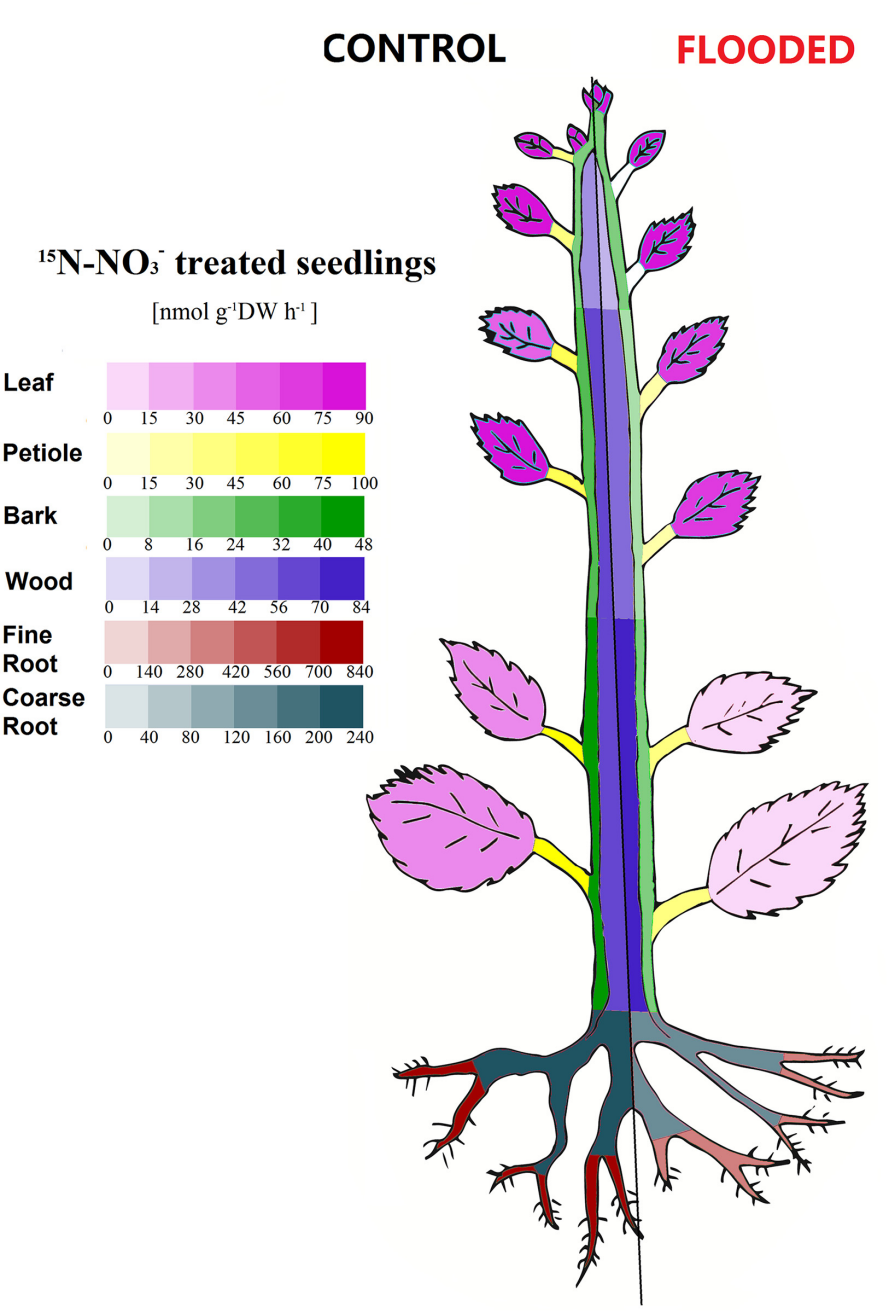
Effect of hypoxia on ^15^N allocation rates of ^15^N derived from ^15^NO_3_^-^ in young poplar trees. Poplar plants were treated as described in legend of Fig 3. ^15^N contents in all plant organs in different plant parts (top 40 cm, middle 40 cm, lowest 50 cm, fine and coarse roots) were determined and data used to calculate ^15^N allocation rates to these organs. The color codes indicate the magnitude of the allocation rates to the organs. Data shown are means ± SD of 10–12 biological replicates. Statistically significant differences between normoxic and hypoxic plants were tested by Student’s t-test and are indicated in S1 Fig.

**Fig 6:**
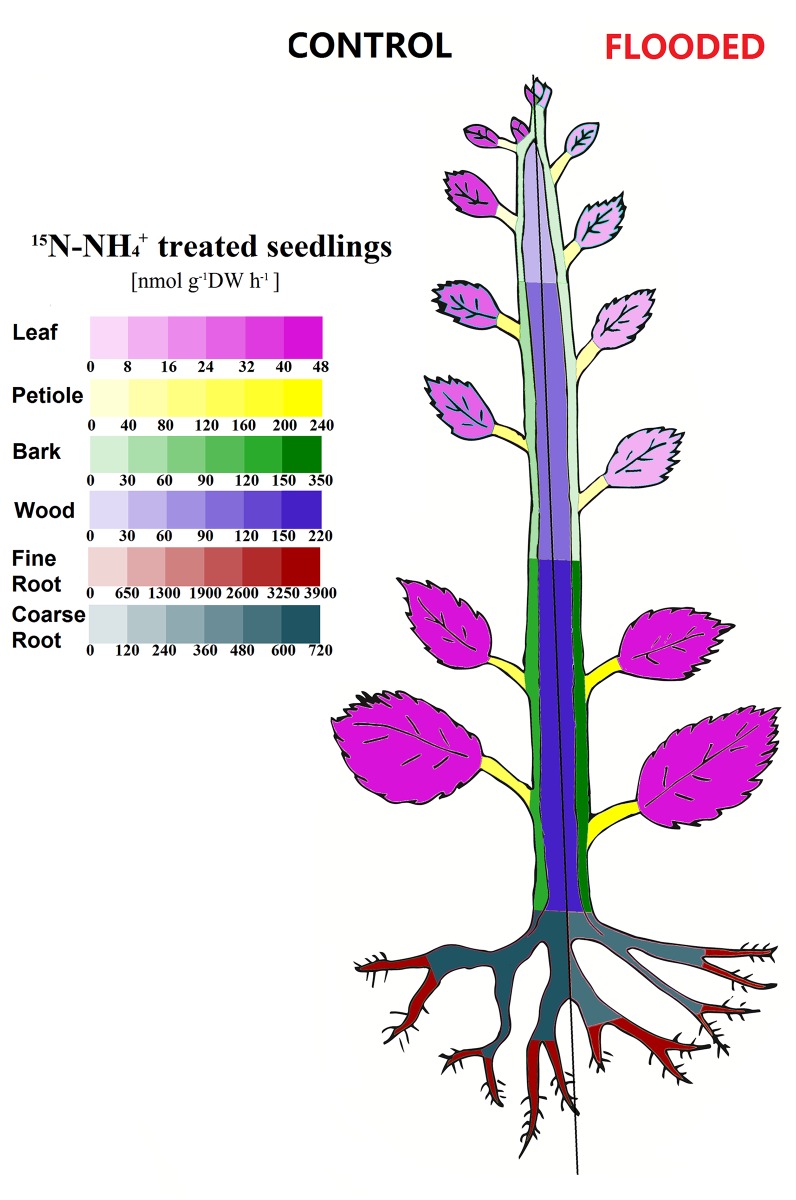
Effect of hypoxia on ^15^N allocation rates of ^15^N derived from ^15^NH_4_^+^ in young poplar trees. Poplar plants were treated as described in legend of Fig 3. ^15^N contents in all plant organs in different plant parts (top 40 cm, middle 40 cm, lowest 50 cm, fine and coarse roots) were determined and data used to calculate ^15^N allocation rates to these organs. The color codes indicate the magnitude of the allocation rates to the organs. Data shown are means ± SD of 10–12 biological replicates. Statistically significant differences between plants exposed to normoxia or hypoxia were tested by Student’s t-test and are indicated in S1 Fig.

We calculated ^15^N allocation rates to the different plant parts of the poplar trees kept under different O_2_ availability (Figs 5 and 6, S1 Fig). As expected from the ^15^N abundance in fine roots, the allocation of ^15^N to this organ dropped due to hypoxia by ca. 50% from 886±205 nmol g^-1^ DW h^-1^ to 411±154 nmol g^-1^ DW h^-1^ (Fig 5). Obviously, the trees allocated major portions of the ^15^N taken up to the developing and young mature leaves at rates of ca. 70–90 nmol g^-1^ DW h^-1^. These allocation rates were independent of the trees’ treatment. However, in contrast to the uppermost plant parts including leaves and bark, which were well supplied with ^15^NO_3_^-^N under soil O_2_ deficiency, allocation rates to petioles and bark dropped in the middle and lowest part of the trees under these conditions.

The effects of O_2_ shortage on the allocation rates of ^15^NH_4_^+^-N clearly differed from that of ^15^NO_3_^-^. There was, for example, no difference between hypoxia and normoxia in the allocation rates into the fine roots (Fig 6), reflecting unaffected ^15^NH_4_^+^ uptake and ^15^N allocation to the shoot under soil O_2_ deficiency. Independent of the O_2_ concentration of the nutrient solution, most of the ^15^NH_4_^+^-N was transported to the older, *i*.*e*. lower, parts of the trees, which was in distinct contrast to the allocation of ^15^NO_3_^-^N. Hypoxia caused significantly lowered allocation rates of ^15^NH_4_^+^-N to the young leaves (hypoxia: 11±7 nmol g^-1^ DW h^-1^; normoxia: 38±25 nmol g^-1^ DW h^-1^) and the petioles of the young mature part of the shoot (hypoxia: 39±26 nmol g^-1^ DW h^-1^; normoxia: 92±49 nmol g^-1^ DW h^-1^). The allocation rates to all other tissues were not significantly affected by soil O_2_ shortage.

## Discussion

Soil O_2_ deprivation strongly impairs mitochondrial respiration causing a cellular energy crisis in the plant tissues affected [17]. As a consequence, ATP consuming processes such as nutrient uptake can be severely impaired [48–50]. In the present study, we focused on plant N metabolism and investigated N uptake and plant internal distribution of currently absorbed N as well as N allocation rates in poplar, a highly flood tolerant, riparian tree species.

### Soil O_2_ deprivation reduces N uptake by poplar roots

In accordance with previous studies on conifers and deciduous trees [6, 12, 51, 52], young poplar trees preferred NH_4_^+^ over NO_3_^-^ as N source. This might be a tree specific feature since herbaceous plants such as rice and maize took up NH_4_^+^ and NO_3_^-^ at similar rates [53]. Interestingly, in this study with crop plants a narrow part of the root (a few mm) directly behind the root tips also preferred NH_4_^+^ over NO_3_^-^. In our study with poplar, NH_4_^+^ was absorbed at ca. 3-times higher rates than NO_3_^-^ under normal O_2_ supply. In accordance to our hypothesis (i), this difference even increased under O_2_ deprivation, because of significantly reduced NO_3_^-^ uptake but unaffected NH_4_^+^ absorption (Fig 3). Thus, although O_2_ levels are not the causal explanation for the difference between NH_4_^+^ and NO_3_^-^ absorption under normal O_2_ supply, reduced soil O_2_ levels seem to exacerbate this situation. Importantly, reduced NO_3_^-^ uptake was not only due (i) to lowered uptake rates on a root fresh weight basis but also (ii) to diminished root biomass (Table 1) enhancing the effects at the whole plant level (Fig 3). Such results are in good agreement with earlier studies indicating reduced NO_3_^-^ absorption by roots of woody species [30, 31, 54]. In contrast, rice plants grown in a low O_2_ root environment did not show reduced NO_3_^-^ uptake most probably because under these conditions structural adaptation prevented O_2_ loss from roots and ensured maintenance of an aerobic metabolism in the roots [55]. The preference of plants to different N sources depends on species and soil properties, for example, soil pH, temperature and abundances of different N forms [56]. The observed preferential absorption of NH_4_^+^ over NO_3_^-^ is often seen in tree species adapted to flood prone environments [30, 57, 58] and might be of ecological advantage, because the energy demand for NH_4_^+^ uptake and assimilation is much lower than for NO_3_^-^ use [59]. On the other hand, in riparian soils NH_4_^+^ is more abundant than NO_3_^-^ during flooding periods [9, 60]. This is, because under such conditions, NO_3_^-^ can be (i) partially converted into NH_4_^+^ by microorganisms, (ii) lost by leaching with flood water or (iii) volatilized and lost as gaseous N (N_2_, N_2_O) due to denitrification [7]. In consistence with the present work, very similar NO_3_^-^ and NH_4_^+^ uptake rates and effects of O_2_ deficiency on N absorption were found in a former study with flooded poplar, where excised roots were supplied with NO_3_^-^ or NH_4_^+^ as the sole N source [31] and not in a combination of the two N sources as in the present study. In good agreement with diminished NO_3_^-^ uptake, considerably reduced transcript levels of NO_3_^-^ transporters were detected in hypoxia treated poplar roots [23].

### Hypoxia affects total N content in poplar roots and shoot but biomass increment only of roots

In the present study, soil O_2_ deficiency caused reduced fine root biomass formation whereas the biomass of the shoot and individual above-ground plant organs remained unaffected (Fig 1, Table 1). Decreased root biomass increment in trees in response to flooding has been observed frequently and was explained by impaired energy metabolism and reduced nutrient uptake [61–66]. Other studies also demonstrated reduced shoot growth which was related to impaired N status of the plants [67]. We assume that in our work the two weeks of soil O_2_ deficiency of this highly flooding tolerant tree species was too short to cause shoot growth reduction. In our study, reduced fine root biomass occurred together with decreased NO_3_^-^ uptake (as expressed on a fresh weight basis); thus, N uptake at the whole plant level considerably decreased under hypoxic conditions (Fig 3). This decline in N absorption was probably responsible for significantly lower N contents in all plant organs of hypoxically treated trees independent on their position on the shoot and regardless of the total amount of N per organ or the relative amount of N per dry weight (Fig 2, Table 2). These results are consistent with previous studies on several plant species including trees where flooding resulted in decreased amounts of total N in plant organs [62, 64, 68]. Such altered concentrations of important nutrients can cause strong nutritional imbalances within plants leading to growth retardation or injury [49]. Diminished leaf N content has been discussed as one reason for reduced rates of photosynthesis [44], which are often observed in flooded trees. Another reason for reduced gas exchange is the closure of stomata [45]; this was most probably also relevant in our study as suggested from the clearly reduced rates of transpiration in hypoxia treated trees compared to controls which supported our hypothesis (ii) (Fig 1).

### The distribution pattern of N derived from NH_4_^+^ and NO_3_^-^ differs in poplar trees

Our results clearly indicated that the main portion of the N taken up remained in the roots, which might partially be due to the experimental procedure to harvest the plants directly after the labelling period. Still, this portion significantly decreased if the roots were exposed to soil O_2_ shortage, *i*.*e*., higher portions of the N taken up were found in the shoot (Fig 4). To obtain a more detailed view of the fate of the NO_3_^-^ and NH_4_^+^ absorbed by the roots, we followed the ^15^N tracer in all plant parts in more detail. For the first time, our study demonstrated that the distribution pattern of N derived from NO_3_^-^ and NH_4_^+^ was different in poplar trees. The highest portion of the ^15^N derived from ^15^NO_3_^-^ was found in the upper parts of the shoot, mainly in the developing and young mature leaves (Table 3). Similar preferential distribution of currently absorbed N to young mature and developing leaves was found in herbaceous plants [69]. Other studies with trees did not differentiate between different developmental stages of plant organs, but also demonstrated that the major portion of ^15^NO_3_^-^ taken up by roots was allocated to the leaves [70, 71, 72]. Besides young leaves, wood of the lower parts of the stem was also a major sink of ^15^N derived from ^15^NO_3_^-^. Interestingly, in contrast to ^15^NO_3_^-^N, the greatest portion of ^15^NH_4_^+^-N was detected in the lowest parts of the stems, namely in bark and wood and only small amounts in the developing leaves. Older leaves of the lower part of the shoot received significant amounts of the ^15^N taken up most probably for incorporation into storage proteins. Soil O_2_ deprivation specifically enhanced this preferential distribution patterns of both ^15^NH_4_^+^-N and ^15^NO_3_^-^N. Such findings seem to be new and similar observations have not been published before. We hypothesize that allocation of different N-forms occurs in a specific manner and speculate that a specific location of transporters mediating xylem unloading of N-compounds exist, which are influenced by the O_2_ availability in the soil [73, 74].

### N allocation rates are specifically altered by soil O_2_ deprivation

Whereas total ^15^N contents in different plant parts indicate the relative distribution of the N absorbed by the plant (Table 3), N allocation rates provide better insight into the processes responsible for this distribution. In the present study we showed for the first time that the allocation rates of NO_3_^-^ and NH_4_^+^ from roots to the shoot were not affected by soil O_2_ availability (Fig 4C). This is astonishing taken into account that the transpiration stream was severely slowed down under these conditions (Fig 1B). To maintain high N allocation rates between roots and the shoot, the xylem sap concentrations of N most probably strongly increased by soil O_2_ shortage. These results suggest that xylem loading of N is not severely impaired by hypoxia and is widely independent of actual uptake rates of NO_3_^-^ and NH_4_^+^.

Highest allocation rates of ^15^NO_3_^-^N were observed to developing and young mature leaves (Fig 5, S1 Fig). We assume that most of the ^15^NO_3_^-^N was transported from root to the shoot in the form of NO_3_^-^. This assumption is indicated from the 10-fold higher *in vivo* NR activity and NR protein abundance in leaves than in roots of poplar trees [37, 38]. It is, therefore, generally assumed that young leaves of poplar are the main site of NO_3_^-^ assimilation. In addition, we observed that a relatively high portion of the ^15^NO_3_^-^N accumulated in the youngest leaves (Table 2) supporting the latter assumption. Surprisingly, soil O_2_ deprivation specifically influenced the allocation rates into individual plant parts. Despite reduced ^15^NO_3_^-^ uptake by roots of hypoxia treated poplar trees, allocation rates of ^15^NO_3_^-^N into developing leaves remained unaffected. However, most organs from middle and lower parts of the shoot received less ^15^NO_3_^-^N under O_2_ shortage. Only the allocation rates to the wood of these shoot sections were unaffected by hypoxia. As bark is considered an important N storage tissue for bark storage proteins (BSP) in poplar [75–77], reduced allocation rates into this tissue might indicate that storage of NO_3_^-^N in the bark was slowed down under O_2_ deprivation. Such changed allocation pattern might be important to maintain N supply to physiologically active tissues in order to enable plant survival during hypoxia.

The allocation rates of ^15^NH_4_^+^-N were completely different from that of ^15^NO_3_^-^N (Fig 6, S1 Fig). Highest ^15^NH_4_^+^-N allocation rates were to the lower—older—plant parts, whereas allocation to the developing parts of the shoot was ca. 3-times slower. Such preferential translocation rates of different N sources to the older parts of the trees have not been described so far; it could be related to the biosynthesis of storage proteins in wood and bark tissue. The mechanism underlying such specific allocation is not clear and should be in the focus of future research. Specific allocation could be a consequence of xylem unloading of reduced N such as amino acids or ^15^NH_4_^+^ [78], or of phloem transport of reduced N from leaves back to these tissues. From the present study it cannot be concluded, if ^15^NH_4_^+^-N was transported in form of NH_4_^+^ or as organic N, for example, as amino acids. Under normal O_2_ supply, NH_4_^+^ taken up is assimilated in the roots yielding glutamine and glutamate [79, 80]. It cannot be excluded that this energy demanding process is inhibited under O_2_ deficiency as also suggested from gene expression data indicating reduced transcript abundance of glutamine synthetase (GS) and NADH-glutamine-oxoglutarate aminotransferase (NADH-GOGAT) in hypoxia treated poplar roots [23]. We therefore assume that the relative portion of NH_4_^+^ which was transported from roots to the shoot increased under hypoxic conditions at the expense of amino acids.

## Conclusion

Taken together, the observed N allocation patterns suggest that plant internal distribution of N is specific regarding the N source taken up by the roots. Moreover, it strongly depends on environmental conditions such as O_2_ supply to the roots. In general, the observed allocation patterns of currently absorbed N derived from both ^15^NO_3_^-^ and ^15^NH_4_^+^ widely reflected the reduced N contents in the different plant organs under hypoxia. Changes in source-sink relations together with changes in xylem unloading processes might be responsible for such findings. Still, further research is needed to elucidate the underlying mechanisms for such compound specific N allocation patterns and the influence of soil O_2_ deprivation on them.

## Supporting Information

S1 Fig Effect of hypoxia on ^15^N allocation rates of ^15^N derived from ^15^NO_3_^-^ (A, B, C, D) and ^15^NH_4_^+^ (E, F, G, H) in young poplar trees. Poplar plants were treated as described in legend of Fig 3. ^15^N contents in all plant organs in different plant parts (A, E: top 40 cm, B, F: middle 40 cm, C, G: lowest 50 cm, D, H: fine and coarse roots) were determined and data used to calculate ^15^N allocation rates to these organs. The color codes indicate the magnitude of the allocation rates to the organs. Data shown are means ± SD of 10–12 biological replicates. Statistically significant differences at p<0.05 between normoxic and hypoxic plants were tested by Student’s t-test and are indicated by asterisks. TL, ML, LL: leaves of top, middle and lowest plant part; TP, MP, LP: petioles of top, middle, lowest part; TB, MB, LB: bark of top, middle, lowest part; TW, MW, LW: wood of top, middle lowest part. FR: fine roots, CR, coarse roots.
(TIF)
